# Results of the Use of Platelet‐Rich Plasma in the Donor Site of Split‐Thickness Skin Grafts: An Exploratory Cohort Study

**DOI:** 10.1111/iwj.70852

**Published:** 2026-03-06

**Authors:** Pedro Fabián Lopez‐Aldana, Juan Darío Alviar Rueda, Jorge Andrés Rueda Gutiérrez, Christian Camilo Tavera‐Sanabria, María Camila Rojas Gómez, Angie Marcela Vargas Duarte, Victoria María Barbosa Tarazona, Sergio Alejandro Gomez‐Ochoa

**Affiliations:** ^1^ Universidad Industrial de Santander Bucaramanga Colombia; ^2^ Hospital Universitario de Santander Bucaramanga Colombia; ^3^ Universidad del Rosario Bogotá D.C Colombia; ^4^ Fundacion Cardiovascular de Colombia Floridablanca Colombia

**Keywords:** graft survival, pain, platelet‐rich plasma, skin transplantation, surgical wound, wound healing

## Abstract

Split‐thickness skin autografts are commonly used to treat extensive cutaneous defects. However, donor site morbidity, including pain, bleeding, and delayed epithelialization, remains a major clinical challenge. This study evaluates whether applying autologous platelet‐rich plasma (PRP) to the donor site improves healing outcomes. A prospective cohort study was conducted at a tertiary‐level academic hospital in Colombia. The study protocol was approved by the local Institutional Ethics Committee. Adult patients (> 18 years) undergoing split‐thickness skin grafts for trauma, burns, oncologic resections, or chronic ulcers were included. Two groups were compared: the PRP group, in which autologous platelet‐rich plasma was applied to the donor site, and the control group, which received standard wound care. The primary outcome was the quality of epithelialization at the donor site, while pain, assessed using the Numeric Rating Scale, was evaluated as a secondary outcome at multiple postoperative time points. Data were analysed using descriptive statistics and linear mixed‐effects models adjusted for potential confounders, with statistical significance set at *p* < 0.05. A total of 46 patients were included (16 in the PRP group and 30 in the control group), with no significant demographic differences between groups. The PRP group demonstrated improved epithelialization quality, with lower Vancouver Scar Scale scores on postoperative days 7 and 14 (*p* < 0.05). Patients treated with PRP also reported a reduction of up to 50% in postoperative pain during early assessments (*p* < 0.001). These effects were maintained throughout the follow‐up period, suggesting a sustained benefit of PRP on both healing quality and pain control. These findings suggest that autologous PRP application at split‐thickness skin graft donor sites may enhance early epithelialization quality and reduce postoperative pain compared with standard wound care. PRP appears to be safe and may represent a useful adjunct to promote improved wound healing and patient recovery in reconstructive surgery. However, larger randomised controlled trials are required to confirm these findings and to establish the clinical effectiveness of autologous PRP in this setting.

## Key Points

- Autologous PRP enhances early donor site epithelization.
- PRP reduces early postoperative pain after skin grafting.
- Sustained short term benefits were observed.
- PRP appears safe as an adjunct in reconstructive surgery.

## 1 Introduction

Split‐thickness skin autografts represent a cornerstone technique in reconstructive surgery, allowing coverage of extensive skin defects resulting from trauma, burns, oncologic resections, and chronic ulcers [1]. Despite their clinical effectiveness, donor sites are associated with considerable morbidity, including pain, bleeding, pruritus, and delayed epithelialization [1, 2]. In many cases, morbidity is greater at the donor site than at the grafted area, significantly affecting patient recovery and quality of life [3].

Platelet‐rich plasma (PRP) is an autologous blood product with platelet concentrations above baseline, enriched with growth factors such as PDGF, TGF‐β, VEGF, and EGF [1]. These bioactive molecules play key roles in haemostasis, angiogenesis, and tissue regeneration. PRP has been increasingly applied in various surgical specialties—including orthopaedics, dermatology, and reconstructive surgery—with encouraging results in wound healing [2, 4]. Preliminary clinical studies suggest that PRP may accelerate epithelialization, reduce pain, and lower complication rates in donor sites of split‐thickness skin grafts [5, 6, 7, 8]. This study aimed to evaluate the effectiveness of PRP in improving donor site healing outcomes, specifically epithelialization time and pain intensity, compared with standard wound care.

## 2 Materials and Methods

A prospective cohort study was conducted at a tertiary‐level academic hospital in Colombia. The study protocol was approved by the local Institutional Ethics Committee. Adult patients over 18 years of age who underwent split‐thickness skin grafting due to trauma, burns, oncologic resections, or chronic ulcers were included. Patients for whom adequate follow‐up could not be ensured were excluded. All participants provided written informed consent prior to enrolment.

Patients were allocated into two treatment groups. In the PRP group, autologous platelet‐rich plasma was obtained using a standardised centrifugation protocol and applied directly to the donor site, followed by placement of a conventional dressing. In the control group, identical standard wound care was provided without PRP application. Skin grafts were harvested by multiple surgeons, and all grafts were obtained at a uniform thickness of 0.4 mm.

Autologous platelet‐rich plasma was prepared for each participant immediately before graft harvesting. A volume of 20–40 mL of peripheral venous blood was collected into tubes containing 3.8% sodium citrate as an anticoagulant. Samples were processed using a two‐step centrifugation method. The first centrifugation (‘soft spin’) was performed at 1500 rpm for 10 min to separate plasma and the buffy coat from red blood cells. The plasma fraction was subsequently subjected to a second centrifugation (‘hard spin’) at 3000 rpm for 10 min to concentrate platelets. The lower one‐third of the plasma fraction, corresponding to the platelet‐rich portion, was collected and used as PRP.

**FIGURE 1:**
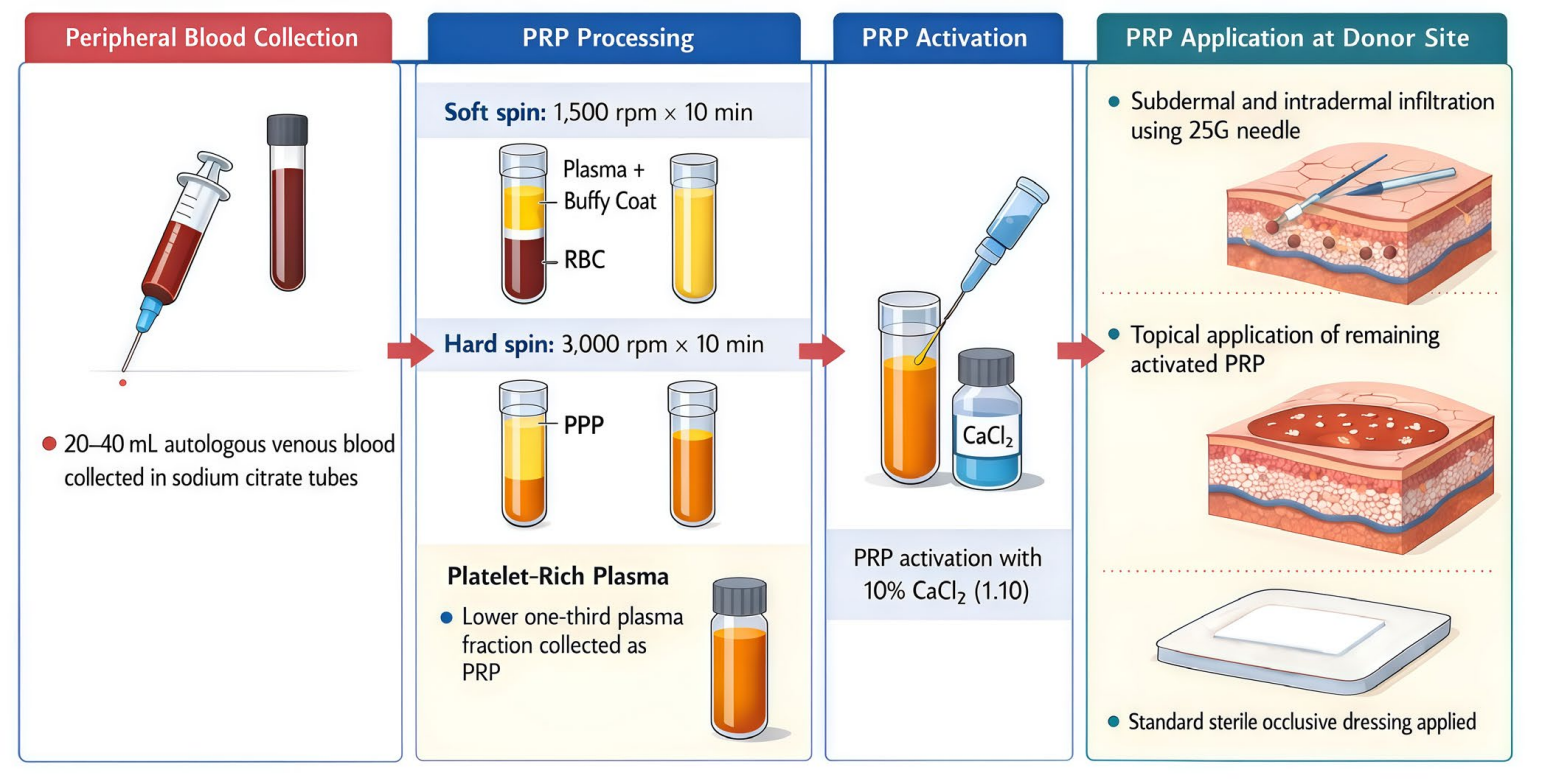
Workflow autologous platelet‐rich plasma (PRP) preparation, activation, and application protocol at the donor site following split‐thickness skin graft harvesting. Peripheral venous blood (20–40 mL) was collected in sodium citrate tubes and processed using a two‐step centrifugation method, including a soft spin (1500 rpm for 10 min) followed by a hard spin (3000 rpm for 10 min) to obtain the platelet‐rich plasma fraction. PRP was activated with 10% calcium chloride (1:10). Following split‐thickness skin graft harvesting, activated PRP was applied to the donor site through subdermal and intradermal infiltration using a 25‐gauge needle, with the remaining PRP applied topically. A standard sterile occlusive dressing was subsequently placed.

**FIGURE 2:**
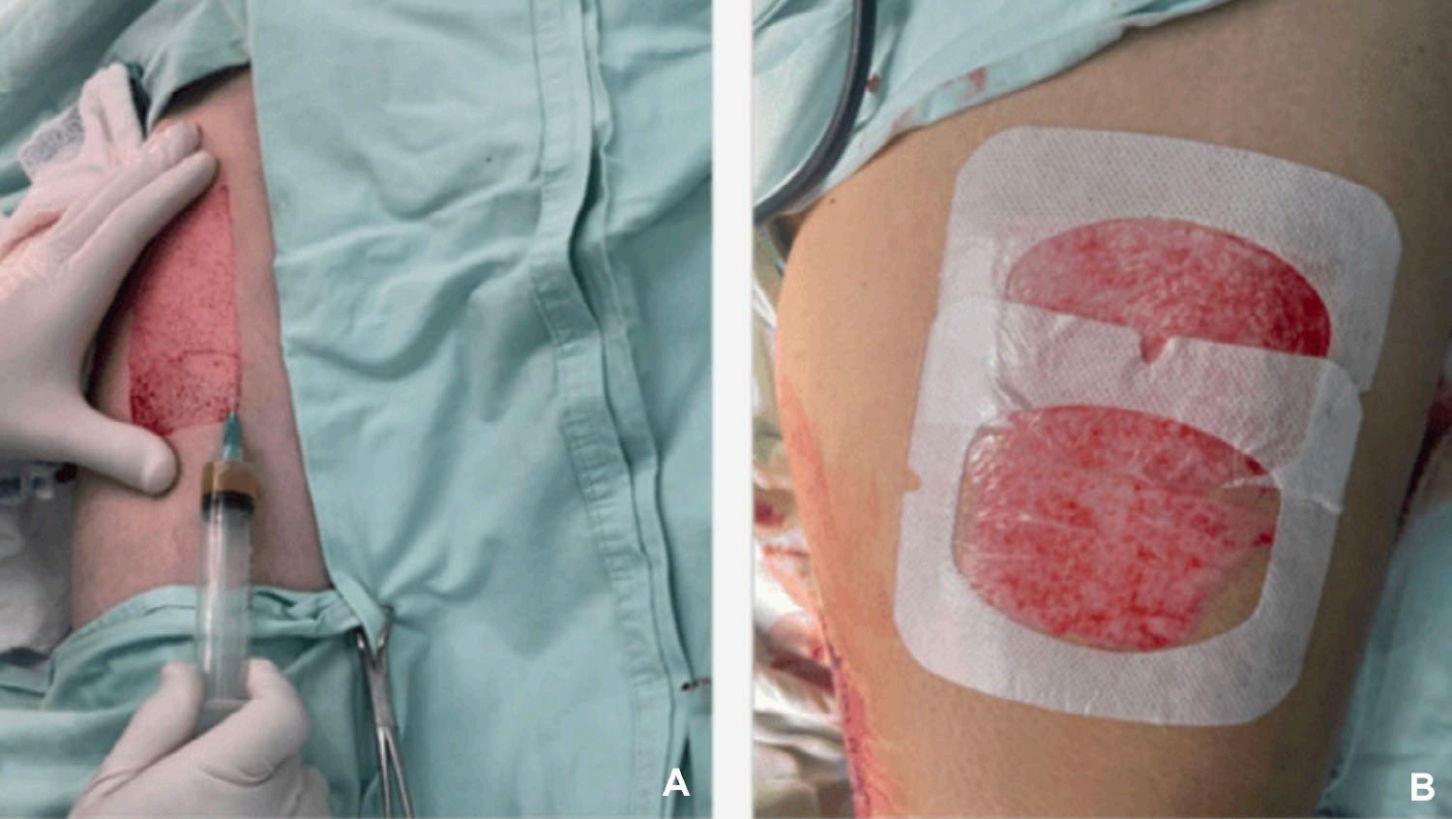
Intraoperative application of platelet‐rich plasma (PRP) at the donor site of a split‐thickness skin graft. (A) Subdermal and intradermal infiltration of activated PRP. (B) Final appearance of the donor site after topical PRP application and placement of a standard sterile occlusive dressing.

Prior to application, PRP was activated with 10% calcium chloride at a 1:10 ratio and gently agitated. After harvesting of the split‐thickness skin graft, PRP was infiltrated into the donor site using a 25‐gauge needle in a combined subdermal and intradermal pattern to ensure uniform distribution. The remaining activated PRP was subsequently applied topically over the donor site surface (see workflow in Figure 1). Standard sterile occlusive dressings were then applied in all cases, following a standardised wound care protocol (see Figure 2).

The primary outcome was the quality of epithelialization of the donor site assessed on postoperative days 7, 14, 21, and 31 using the Vancouver Scar Scale [9]. Secondary outcomes included pain intensity, measured with the Numeric Rating Scale (visual analog score) from 0 to 10 on postoperative days 7 and 14 [10].

Descriptive statistics were used to summarise baseline patient demographics and clinical characteristics. Continuous variables were presented as mean ± standard deviation (SD) or median and interquartile range (IQR), while categorical variables were presented as counts and percentages. Baseline group comparisons were made using the Kruskal–Wallis test for continuous variables and the Chi‐square test or Fisher's exact test for categorical variables, as appropriate.

To account for the repeated‐measures design and intra‐patient correlation in longitudinal outcomes (Vancouver Scar Scale and pain scores), linear mixed‐effects models (LMMs) were employed. The models included fixed effects for the treatment group (PRP vs. Control), time (continuous), and the group‐by‐time interaction. A random intercept for each patient was included to account for baseline variability, and a random slope for time was included to allow for individual differences in healing trajectories. This random structure was selected as the optimal fit based on a lower Akaike Information Criterion (AIC) than a random‐intercept‐only model and a model assuming a compound symmetry covariance structure.

The final models were fitted using Restricted Maximum Likelihood (REML). Model assumptions, including normality and homoscedasticity of residuals, were assessed visually using QQ‐plots and residuals‐versus‐fitted plots. Estimated marginal means (EMMs) were calculated from the final models to provide adjusted group means at each assessment time point. Pairwise comparisons of EMMs between the PRP and Control groups were conducted at each time point, with *p*‐values adjusted for multiple comparisons using the False Discovery Rate (FDR) method.

An additional exploratory analysis was performed using a cumulative link mixed model (CLMM) to appropriately model the ordinal Vancouver scores. A final multivariable LMM, adjusted for potential baseline confounders including age, aetiology, and comorbidities, was also fitted to assess the robustness of the primary findings. All statistical analyses were performed using R version 4.3.2 (R Foundation for Statistical Computing, Vienna, Austria). A two‐tailed *p*‐value < 0.05 was considered statistically significant for all analyses.

## 3 Results

### 3.1 Patient Characteristics

A total of 46 patients who underwent split‐thickness skin grafting were included in the analysis. Of these, 16 patients received PRP treatment and 30 received standard wound care (control group). No statistically significant differences were observed between groups regarding baseline demographic characteristics, clinical variables, or surgical parameters. Both groups demonstrated comparable age (mean, 44.0 vs. 37.3 years; *p* = 0.137) and sex distributions (73.3% vs. 62.5% male; *p* = 0.447), as summarised in Table 1.

**TABLE 1:** Baseline patient demographics and clinical characteristics.

	Control (*N* = 30)	PRP (*N* = 16)	Total (*N* = 46)	*p*
Age				0.137
Mean (SD)	44.0 (14.872)	37.312 (14.541)	41.674 (14.946)	
Median (Q1, Q3)	43.0 (31.8, 54.0)	35.500 (26.0, 52.5)	41.0 (29.3, 54.0)	
Range	18.0–73.0	16.0–62.0	16.0–73.0	
Age group				0.341
≤ 40	13 (43.3%)	10 (62.5%)	23 (50.0%)	
41–60	11 (36.7%)	5 (31.2%)	16 (34.8%)	
> 60	6 (20.0%)	1 (6.2%)	7 (15.2%)	
Sex				0.447
Male	22 (73.3%)	10 (62.5%)	32 (69.6%)	
Female	8 (26.7%)	6 (37.5%)	14 (30.4%)	
Nationality				0.462
Colombiano	26 (86.7%)	15 (93.8%)	41 (89.1%)	
Venezolano	4 (13.3%)	1 (6.2%)	5 (10.9%)	
Area of residence				0.497
Rural	5 (16.7%)	4 (25.0%)	9 (19.6%)	
Urban	25 (83.3%)	12 (75.0%)	37 (80.4%)	
Education				
None	6	2	8	
Primary	13 (54.2%)	7 (50.0%)	20 (52.6%)	
High school	11 (45.8%)	7 (50.0%)	18 (47.4%)	
Technical	0 (0.0%)	0 (0.0%)	0 (0.0%)	
University	0 (0.0%)	0 (0.0%)	0 (0.0%)	
Insurance type				0.487
Contributory	1 (3.3%)	2 (12.5%)	3 (6.5%)	
Private	2 (6.7%)	0 (0.0%)	2 (4.3%)	
Soat (traffic accident insurance)	11 (36.7%)	6 (37.5%)	17 (37.0%)	
Subsidised	16 (53.3%)	8 (50.0%)	24 (52.2%)	
Nutritional status				0.828
Underweight (BMI < 18.5)	2 (8.7%)	1 (7.1%)	3 (8.1%)	
Normal (IMC 18.5–24.9)	16 (69.6%)	11 (78.6%)	27 (73.0%)	
Overweight (BMI 25.1–29.9)	5 (21.7%)	2 (14.3%)	7 (18.9%)	
Albumin				
< 3.5	0 (0.0%)	0 (0.0%)	0 (0.0%)	
> 3.5	11 (68.8%)	7 (58.3%)	18 (64.3%)	
Exam not performed	5 (31.2%)	5 (41.7%)	10 (35.7%)	
Graft length (cm)				0.186
Mean (SD)	4.267 (0.785)	3.938 (0.680)	4.152 (0.759)	
Median (Q1, Q3)	4.0 (4.0, 5.0)	4.0 (3.750, 4.0)	4.0 (4.0, 5.0)	
Range	3.0–6.0	3.0–5.0	3.0–6.0	
Aetiology				0.873
Burns	9 (30.0%)	6 (37.5%)	15 (32.6%)	
Necrotizing fasciitis	4 (13.3%)	2 (12.5%)	6 (13.0%)	
Trauma	17 (56.7%)	8 (50.0%)	25 (54.3%)	
Hospital stay				0.455
Mean (DE)	65.759 (63.4)	45.500 (25.3)	58.556 (53.6)	
Median (Q1, Q3)	45.0 (31.0, 56.0)	38.0 (29.8, 51.0)	40.0 (31.0, 56.0)	
Range	19.0–269.0	6.0–92.0	6.0–269.0	
Prolonged hospitalisation				0.608
No	14 (48.3%)	9 (56.2%)	23 (51.1%)	
Si	15 (51.7%)	7 (43.8%)	22 (48.9%)	
Comorbidities				0.956
No	19 (63.3%)	10 (62.5%)	29 (63.0%)	
Si	11 (36.7%)	6 (37.5%)	17 (37.0%)	

### 3.2 Primary Outcome: Donor Site Healing Quality (Vancouver Scar Scale)

A significant main effect of treatment group was observed, indicating improved donor site healing quality in patients treated with PRP compared with the control group. At the first postoperative assessment (day 7), the PRP group demonstrated a lower (more favourable) estimated marginal mean (EMM) Vancouver Scar Scale score of 4.70 (95% CI, 4.32–5.08), compared with 5.25 (95% CI, 4.97–5.53) in the control group. This corresponded to a mean difference of 0.55 points, which was statistically significant (*p* = 0.024).

This benefit was sustained at subsequent assessment time points. At postoperative day 14, the estimated marginal mean Vancouver Scar Scale score was 3.23 (95% CI, 2.94–3.51) in the PRP group, compared with 3.69 (95% CI, 3.48–3.90) in the control group (*p* = 0.012). At postoperative days 21–31, mean scores were 2.49 (95% CI, 2.14–2.84) for the PRP group and 2.91 (95% CI, 2.66–3.17) for the control group (*p* = 0.055). After adjustment for multiple comparisons, between‐group differences remained statistically significant at postoperative days 7 and 14 (false discovery rate–adjusted *p* < 0.05).

**FIGURE 3:**
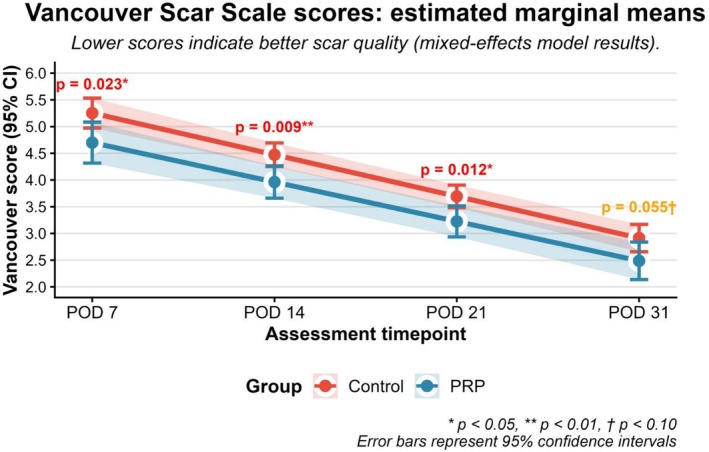
Healing trajectories of donor sites based on Vancouver Scar Scale scores. The plot displays the model‐adjusted estimated marginal means with 95% confidence intervals. The PRP group demonstrates consistently lower (better) scores at all timepoints.

**FIGURE 4:**
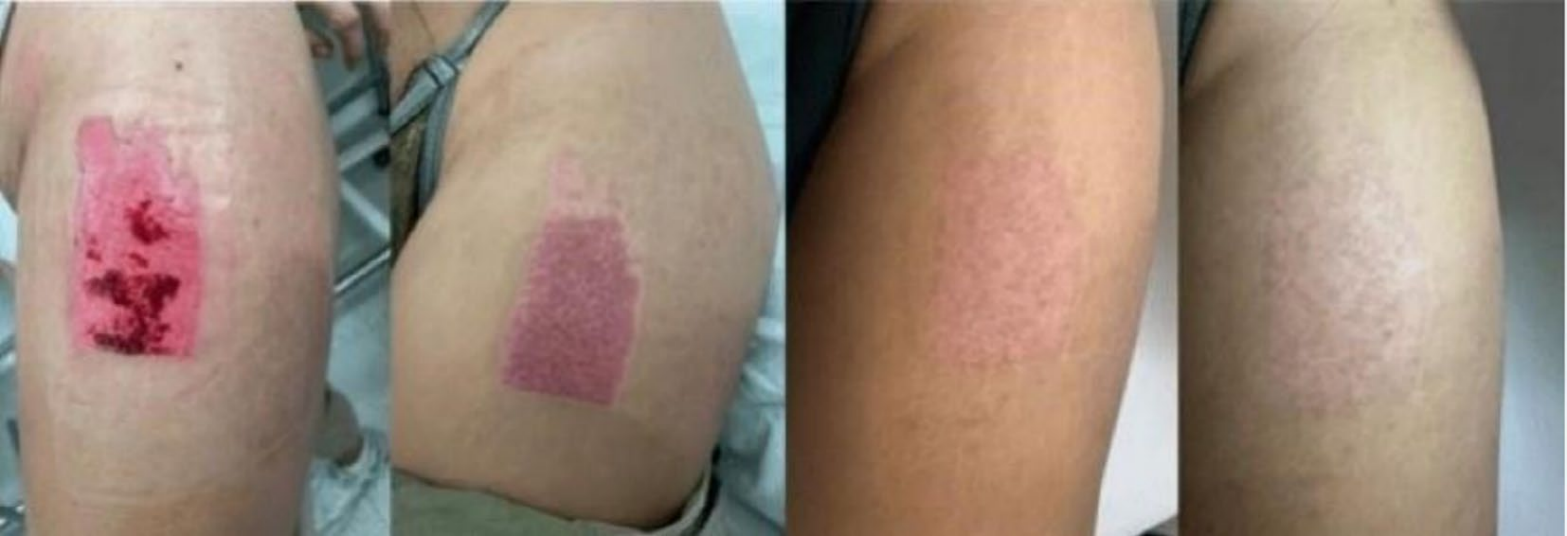
Representative clinical photographs of the split‐thickness skin graft donor site in a patient treated with platelet‐rich plasma (PRP), demonstrating progressive healing and scar maturation at postoperative day 7, day 14, day 21, and day 31.

The treatment‐by‐time interaction term was not statistically significant (*β* = 0.043, *p* = 0.449), indicating no difference in the rate of score change over time between groups (Figure 3). A representative clinical case illustrating the macroscopic progression of donor‐site healing over time in a patient treated with PRP is presented in Figure 4.

### 3.3 Secondary Outcomes

**FIGURE 5:**
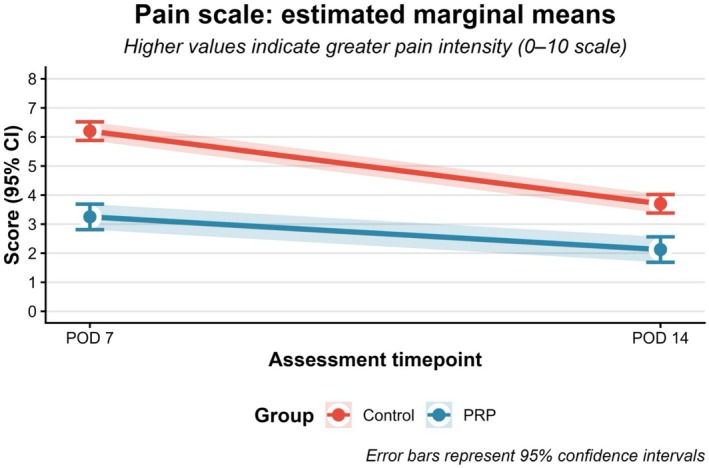
Evolution of postoperative pain intensity between treatment groups.

PRP application was associated with significantly lower postoperative pain scores, with a statistically significant treatment‐by‐time interaction (*p* < 0.001), indicating differential pain trajectories between groups over time (Figure 5).

At postoperative day 7, patients in the PRP group reported lower pain scores compared with the control group. The estimated marginal mean (EMM) pain score in the PRP group was 3.25 (95% CI, 2.81–3.69) on a 10‐point numeric rating scale, whereas the control group demonstrated a mean score of 6.20 (95% CI, 5.88–6.52). This corresponded to a mean between‐group difference of 2.95 points, which was statistically significant (*p* < 0.001).

Although pain scores decreased over time in both groups, statistically significant differences persisted at subsequent follow‐up assessments. The PRP group continued to report lower pain scores, with an EMM of 2.12 (95% CI, 1.69–2.56), compared with 3.70 (95% CI, 3.38–4.02) in the control group, corresponding to a mean difference of 1.57 points (*p* < 0.001).

## 4 Discussion

Split‐thickness skin grafts are widely used in plastic and reconstructive surgery for the management of defects resulting from oncologic resections, burns, trauma, and chronic wounds [1]. Postoperative pain at the donor site remains one of the most frequent patient complaints, prompting ongoing investigation into dressing strategies that may accelerate healing and reduce postoperative discomfort [1, 4, 11].

Platelet‐rich plasma (PRP) has emerged as a biological adjunct for donor‐site management. PRP is an autologous blood‐derived product containing a high concentration of platelets and bioactive growth factors capable of stimulating angiogenesis, cell proliferation, and tissue regeneration. Its application to skin graft donor sites has been proposed as a strategy to enhance wound healing, reduce pruritus and postoperative pain, and improve the quality of the regenerated tissue [1, 2, 4, 5, 12, 13].

Several randomised controlled trials have reported favourable outcomes with PRP, including a reduction in time to epithelialization when compared with conventional wound care. In the present analysis, however, no statistically significant difference in the rate of healing was observed between PRP‐treated and control donor sites, as reflected by the non‐significant treatment‐by‐time interaction (*p* = 0.449). This finding suggests that PRP may not accelerate the overall healing trajectory but rather exerts its primary effect during the early postoperative phase [13, 14].

In agreement with previous reports by Dhua and Chigurupati [6, 15], PRP‐treated donor sites demonstrated lower postoperative pain and reduced pruritus. In addition, improved scar quality was observed, as evidenced by lower Vancouver Scar Scale scores (*p* = 0.024). These findings are consistent with those reported by García‐Sánchez et al. [7], who described superior Vancouver and POSAS scores in donor sites managed with PRP [16]. Collectively, these results support the concept that PRP may contribute to improved qualitative aspects of wound healing, including scar maturation and patient comfort, even in the absence of a measurable acceleration in epithelialization.

Furthermore, a meta‐analysis of six studies published in 2021 demonstrated that PRP significantly reduced healing time (7–14 days vs. 14–18 days; *p* < 0.001) and postoperative pain during dressing changes, without an associated increase in complication rates [4]. Although a shorter healing time was not observed in the present analysis, the improvement in postoperative comfort and scar quality observed supports a potential beneficial role of PRP in donor‐site management.

Several limitations should be acknowledged. First, the relatively small sample size may have limited the statistical power to detect more subtle between‐group differences. Second, although linear mixed‐effects modelling was used to partially account for intra‐individual variability, graft harvesting and donor‐site management were performed by multiple surgeons, which may have introduced technical heterogeneity. Finally, wound healing and pain outcomes were assessed using clinical rating scales that inherently include subjective components, which may have influenced outcome assessment.

Taken together, the findings of this study suggest that PRP may exert its primary effects through modulation of the inflammatory response and stimulation of epithelial regeneration, contributing to improved scar quality and enhanced patient comfort rather than accelerating complete wound closure.

The reduction in postoperative pain observed in the PRP group may be explained by the anti‐inflammatory properties of platelet‐derived growth factors, which are known to modulate cytokine release and reduce local inflammatory mediators such as interleukin‐1 and tumour necrosis factor‐alpha [1, 2, 5, 6, 17]. In addition, the fibrin matrix formed following PRP activation may function as a biological dressing, providing coverage of exposed nerve endings and reducing mechanical irritation during dressing changes [6, 12, 15]. Improved scar quality may also be related to enhanced collagen organisation and more balanced fibroblast activity during the remodelling phase of wound healing [4, 7, 14].

Despite the aforementioned limitations, the present findings suggest that PRP may represent a safe and potentially useful adjunct for donor‐site management. Future studies with larger sample sizes and standardised PRP preparation protocols are warranted to further evaluate these observations and to determine optimal platelet concentrations and timing of application. If confirmed in adequately powered randomised controlled trials, the incorporation of PRP into donor‐site management strategies could offer a feasible and low‐cost approach to improving patient comfort and scar‐related outcomes in reconstructive surgery [2, 4].

## 5 Conclusions

The findings of this study suggest that the application of autologous PRP at donor sites of split‐thickness skin grafts is associated with improved early epithelialization quality and reduced postoperative pain when compared with standard wound care. Patients in the PRP group demonstrated lower Vancouver Scar Scale and Numeric Pain Scale scores at early and intermediate postoperative assessments, reflecting more favourable early wound‐healing characteristics and greater patient comfort. These benefits were maintained throughout the short‐term follow‐up period, indicating a sustained advantage in early healing progression rather than an acceleration of the healing rate. Among the evaluated parameters of early epithelialization and pain control, PRP appeared to be a safe and potentially beneficial adjunct in donor‐site management. Further randomised studies with larger sample sizes and longer follow‐up are required to evaluate long‐term scar maturation and to better define the role of PRP in reconstructive surgery.

## Funding

The authors have nothing to report.

## Ethics Statement

Following approval of our Institutional Review Board and ethical committee, all procedures performed in studies involving human participants were in accordance with the ethical standards of the institutional and/or national research committee and with the 1964 Helsinki Declaration and its later amendments or comparable ethical standards. Informed consent was obtained from all individual participants included in the study.

## Conflicts of Interest

The authors declare no conflicts of interest.

## Data Availability

The data that support the findings of this study are available from the corresponding author upon reasonable request.
